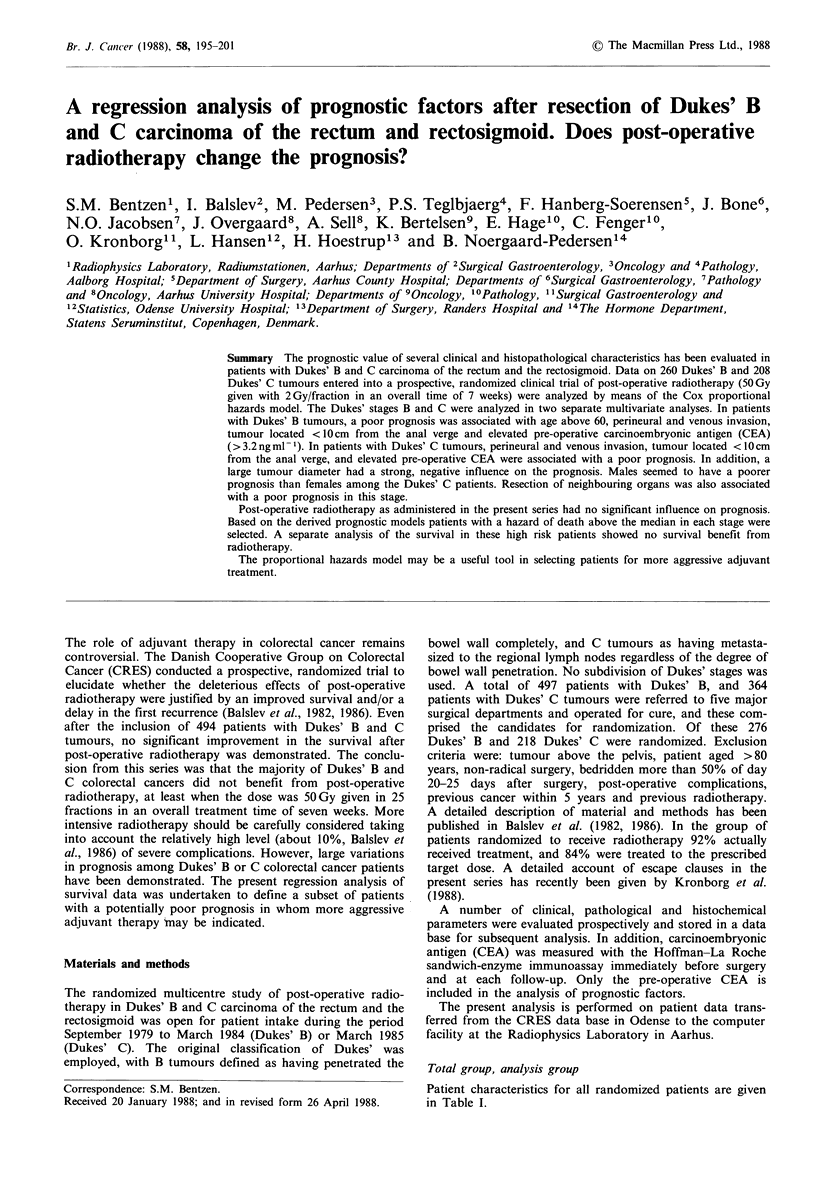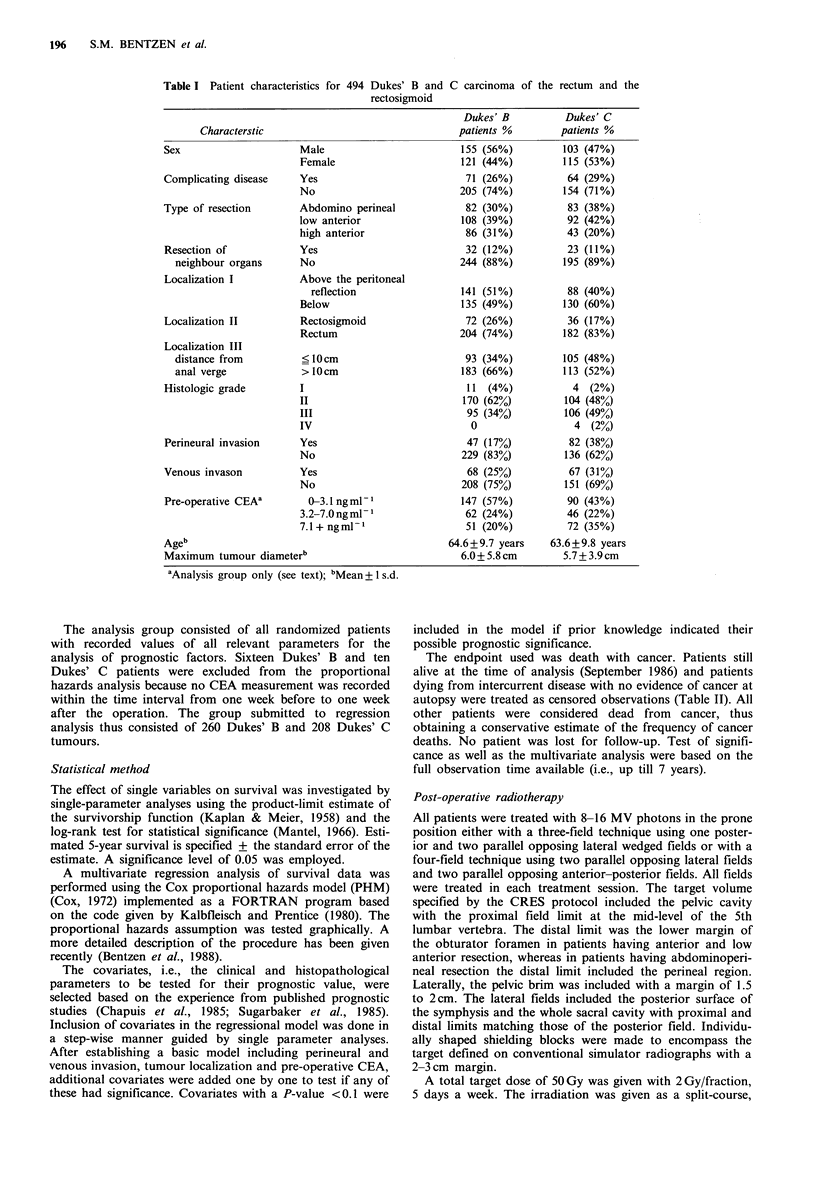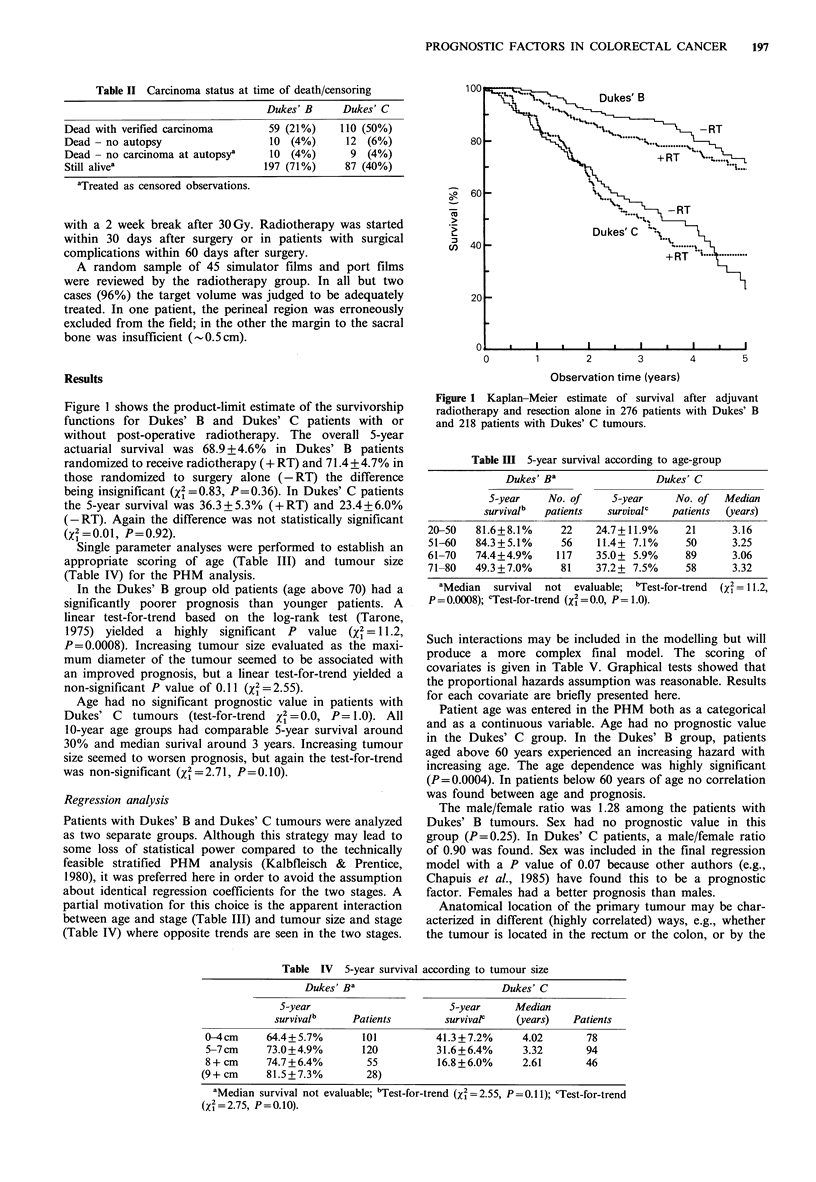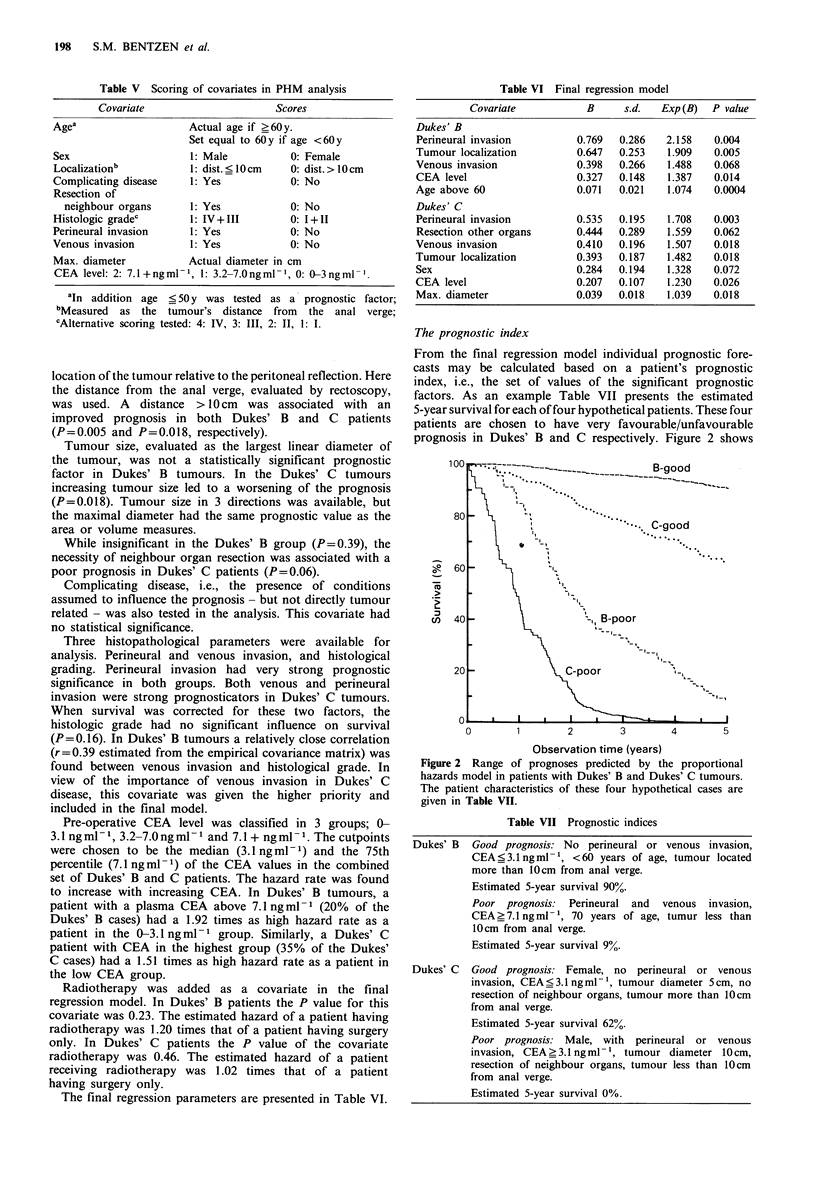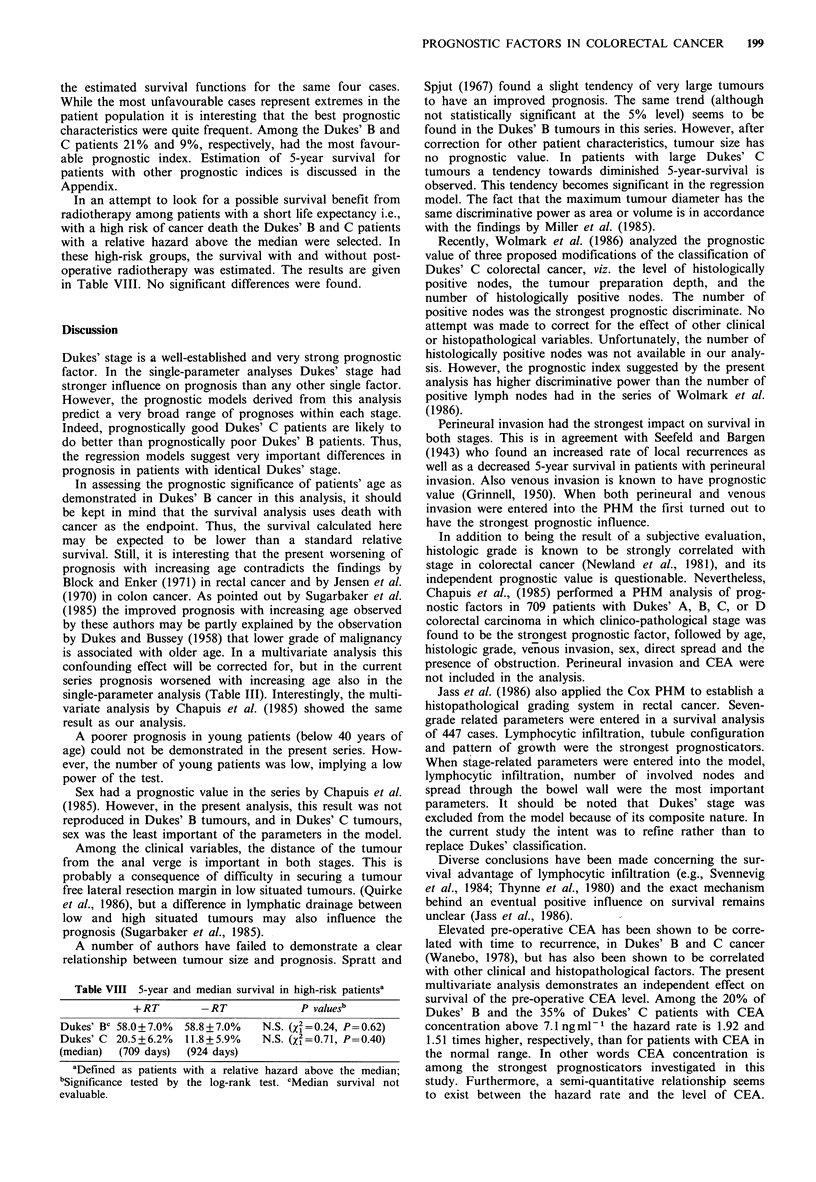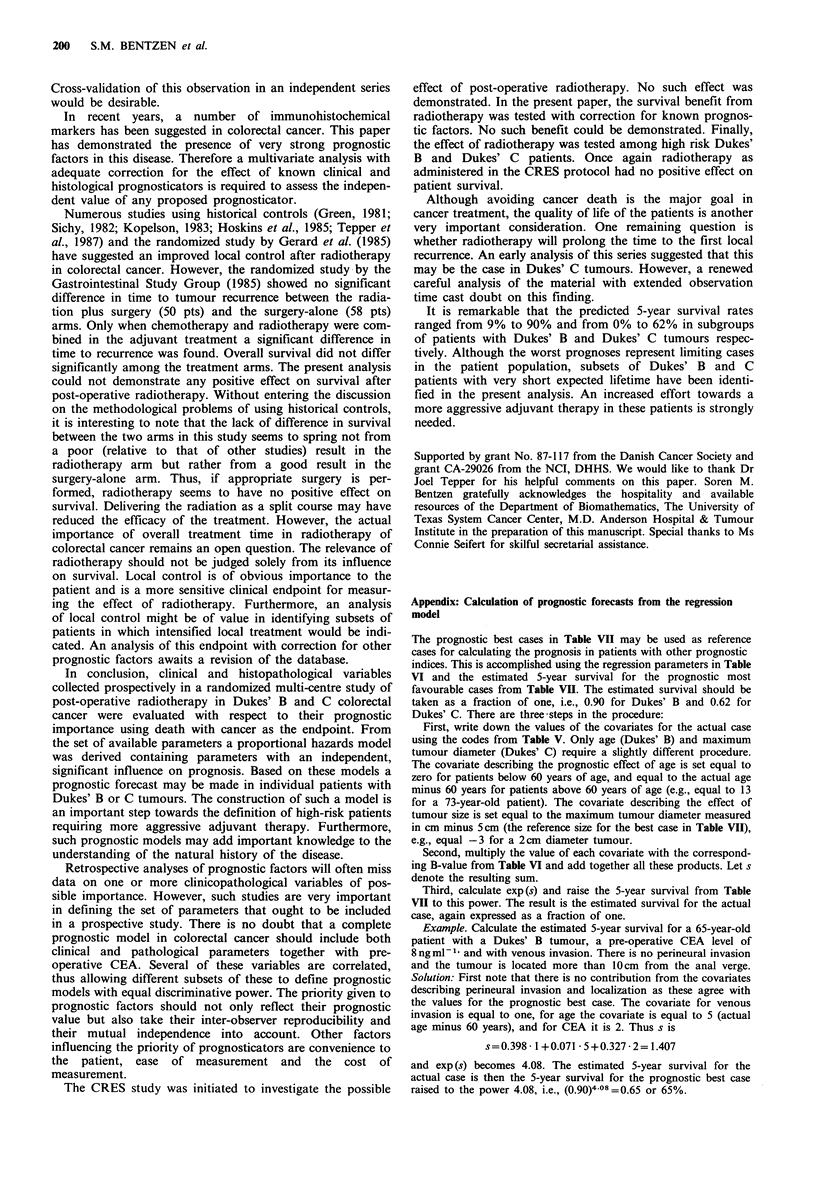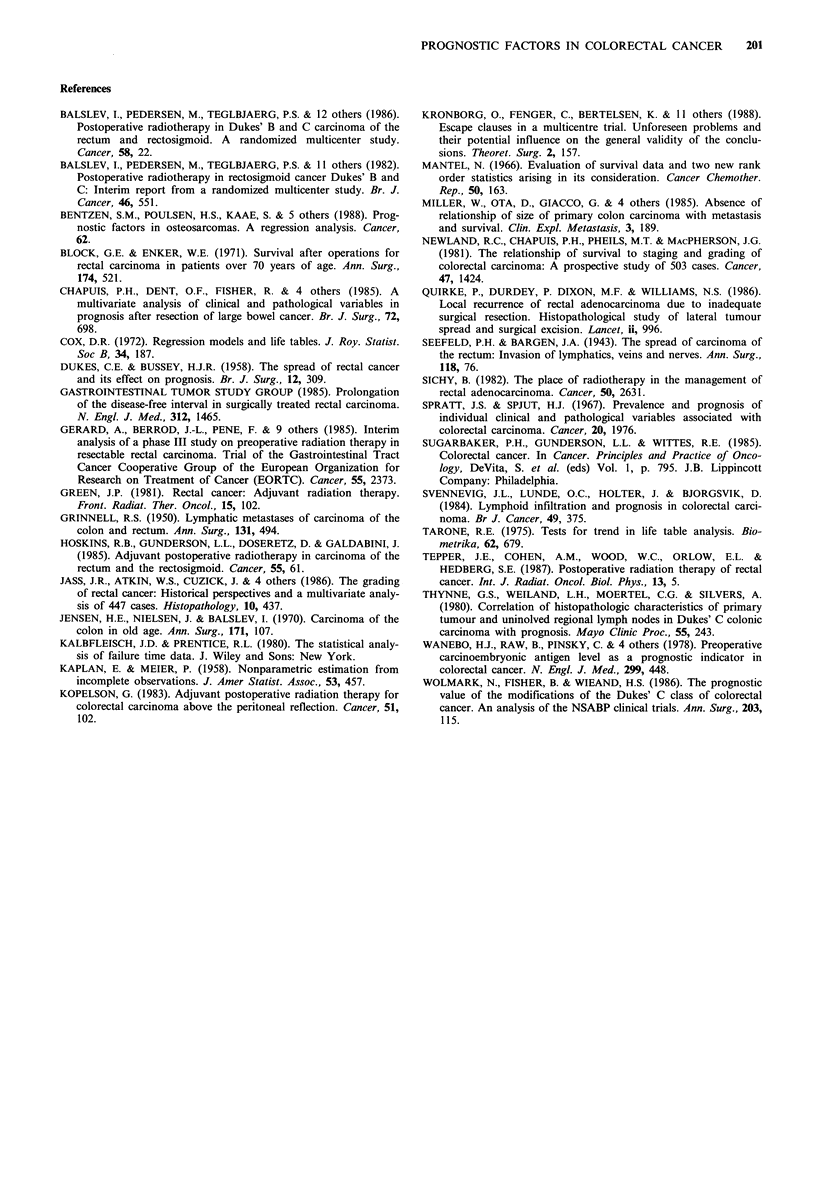# A regression analysis of prognostic factors after resection of Dukes' B and C carcinoma of the rectum and rectosigmoid. Does post-operative radiotherapy change the prognosis?

**DOI:** 10.1038/bjc.1988.192

**Published:** 1988-08

**Authors:** S. M. Bentzen, I. Balslev, M. Pedersen, P. S. Teglbjaerg, F. Hanberg-Soerensen, J. Bone, N. O. Jacobsen, J. Overgaard, A. Sell, K. Bertelsen

**Affiliations:** Radiophysics Laboratory, Radiumstationen, Aarhus.

## Abstract

The prognostic value of several clinical and histopathological characteristics has been evaluated in patients with Dukes' B and C carcinoma of the rectum and the rectosigmoid. Data on 260 Dukes' B and 208 Dukes' C tumours entered into a prospective, randomized clinical trial of post-operative radiotherapy (50 Gy given with 2 Gy/fraction in an overall time of 7 weeks) were analyzed by means of the Cox proportional hazards model. The Dukes' stages B and C were analyzed in two separate multivariate analyses. In patients with Dukes' B tumours, a poor prognosis was associated with age above 60, perineural and venous invasion, tumour located less than 10 cm from the anal verge and elevated pre-operative carcinoembryonic antigen (CEA) (greater than 3.2 ng ml-1). In patients with Dukes' C tumours, perineural and venous invasion, tumour located less than 10 cm from the anal verge, and elevated pre-operative CEA were associated with a poor prognosis. In addition, a large tumour diameter had a strong, negative influence on the prognosis. Males seemed to have a poorer prognosis than females among the Dukes' C patients. Resection of neighbouring organs was also associated with a poor prognosis in this stage. Post-operative radiotherapy as administered in the present series had no significant influence on prognosis. Based on the derived prognostic models patients with a hazard of death above the median in each stage were selected. A separate analysis of the survival in these high risk patients showed no survival benefit from radiotherapy. The proportional hazards model may be a useful tool in selecting patients for more aggressive adjuvant treatment.


Br. J. Cancer (1988), 58, 195-201

© The Macmillan Press Ltd., 1988

### A regression analysis of prognostic factors after resection of Dukes' B and C carcinoma of the rectum and rectosigmoid. Does post-operative radiotherapy change the prognosis?

S.M. Bentzen[1], I. Balslev[2], M. Pedersen[3], P.S. Teglbjaerg[4], F. Hanberg-Soerensen[5], J. Bone[6], N.O. Jacobsen[7], J. Overgaard[8], A. Sell[8], K. Bertelsen[9], E. Hage[10], C. Fenger[10], O. Kronborg[11], L. Hansen[12], H. Hoestrup[13] and B. Noergaard-Pedersen[14]

[1]*Radiophysics Laboratory, Radiumstationen, Aarhus; Departments of* [2]*Surgical Gastroenterology,* [3]*Oncology and* [4]*Pathology, Aalborg Hospital;* [5]*Department of Surgery, Aarhus County Hospital; Departments of* [6]*Surgical Gastroenterology,* [7]*Pathology and* [8]*Oncology, Aarhus University Hospital; Departments of* [9]*Oncology,* [10]*Pathology,* [11]*Surgical Gastroenterology and* [12]*Statistics, Odense University Hospital;* [13]*Department of Surgery, Randers Hospital and* [14]*The Hormone Department, Statens Seruminstitut, Copenhagen, Denmark.*

**Summary** The prognostic value of several clinical and histopathological characteristics has been evaluated in patients with Dukes' B and C carcinoma of the rectum and the rectosigmoid. Data on 260 Dukes' B and 208 Dukes' C tumours entered into a prospective, randomized clinical trial of post-operative radiotherapy (50 Gy given with 2 Gy/fraction in an overall time of 7 weeks) were analyzed by means of the Cox proportional hazards model. The Dukes' stages B and C were analyzed in two separate multivariate analyses. In patients with Dukes' B tumours, a poor prognosis was associated with age above 60, perineural and venous invasion, tumour located $<10$ cm from the anal verge and elevated pre-operative carcinoembryonic antigen (CEA) ($>3.2$ ng ml$^{-1}$). In patients with Dukes' C tumours, perineural and venous invasion, tumour located $<10$ cm from the anal verge, and elevated pre-operative CEA were associated with a poor prognosis. In addition, a large tumour diameter had a strong, negative influence on the prognosis. Males seemed to have a poorer prognosis than females among the Dukes' C patients. Resection of neighbouring organs was also associated with a poor prognosis in this stage.

Post-operative radiotherapy as administered in the present series had no significant influence on prognosis. Based on the derived prognostic models patients with a hazard of death above the median in each stage were selected. A separate analysis of the survival in these high risk patients showed no survival benefit from radiotherapy.

The proportional hazards model may be a useful tool in selecting patients for more aggressive adjuvant treatment.

The role of adjuvant therapy in colorectal cancer remains controversial. The Danish Cooperative Group on Colorectal Cancer (CRES) conducted a prospective, randomized trial to elucidate whether the deleterious effects of post-operative radiotherapy were justified by an improved survival and/or a delay in the first recurrence (Balslev *et al.*, 1982, 1986). Even after the inclusion of 494 patients with Dukes' B and C tumours, no significant improvement in the survival after post-operative radiotherapy was demonstrated. The conclusion from this series was that the majority of Dukes' B and C colorectal cancers did not benefit from post-operative radiotherapy, at least when the dose was 50 Gy given in 25 fractions in an overall treatment time of seven weeks. More intensive radiotherapy should be carefully considered taking into account the relatively high level (about 10%, Balslev *et al.*, 1986) of severe complications. However, large variations in prognosis among Dukes' B or C colorectal cancer patients have been demonstrated. The present regression analysis of survival data was undertaken to define a subset of patients with a potentially poor prognosis in whom more aggressive adjuvant therapy may be indicated.

### Materials and methods

The randomized multicentre study of post-operative radiotherapy in Dukes' B and C carcinoma of the rectum and the rectosigmoid was open for patient intake during the period September 1979 to March 1984 (Dukes' B) or March 1985 (Dukes' C). The original classification of Dukes' was employed, with B tumours defined as having penetrated the bowel wall completely, and C tumours as having metastasized to the regional lymph nodes regardless of the degree of bowel wall penetration. No subdivision of Dukes' stages was used. A total of 497 patients with Dukes' B, and 364 patients with Dukes' C tumours were referred to five major surgical departments and operated for cure, and these comprised the candidates for randomization. Of these 276 Dukes' B and 218 Dukes' C were randomized. Exclusion criteria were: tumour above the pelvis, patient aged $>80$ years, non-radical surgery, bedridden more than 50% of day 20–25 days after surgery, post-operative complications, previous cancer within 5 years and previous radiotherapy. A detailed description of material and methods has been published in Balslev *et al.* (1982, 1986). In the group of patients randomized to receive radiotherapy 92% actually received treatment, and 84% were treated to the prescribed target dose. A detailed account of escape clauses in the present series has recently been given by Kronborg *et al.* (1988).

A number of clinical, pathological and histochemical parameters were evaluated prospectively and stored in a data base for subsequent analysis. In addition, carcinoembryonic antigen (CEA) was measured with the Hoffman–La Roche sandwich-enzyme immunoassay immediately before surgery and at each follow-up. Only the pre-operative CEA is included in the analysis of prognostic factors.

The present analysis is performed on patient data transferred from the CRES data base in Odense to the computer facility at the Radiophysics Laboratory in Aarhus.

### *Total group, analysis group*

Patient characteristics for all randomized patients are given in Table I.

Correspondence: S.M. Bentzen.

Received 20 January 1988; and in revised form 26 April 1988.

**Table I** Patient characteristics for 494 Dukes' B and C carcinoma of the rectum and the rectosigmoid

| *Characterstic* | | *Dukes' B patients %* | *Dukes' C patients %* |
|---|---|---|---|
| Sex | Male | 155 (56%) | 103 (47%) |
| | Female | 121 (44%) | 115 (53%) |
| Complicating disease | Yes | 71 (26%) | 64 (29%) |
| | No | 205 (74%) | 154 (71%) |
| Type of resection | Abdomino perineal | 82 (30%) | 83 (38%) |
| | low anterior | 108 (39%) | 92 (42%) |
| | high anterior | 86 (31%) | 43 (20%) |
| Resection of neighbour organs | Yes | 32 (12%) | 23 (11%) |
| | No | 244 (88%) | 195 (89%) |
| Localization I | Above the peritoneal reflection | 141 (51%) | 88 (40%) |
| | Below | 135 (49%) | 130 (60%) |
| Localization II | Rectosigmoid | 72 (26%) | 36 (17%) |
| | Rectum | 204 (74%) | 182 (83%) |
| Localization III distance from anal verge | ≦10 cm | 93 (34%) | 105 (48%) |
| | >10 cm | 183 (66%) | 113 (52%) |
| Histologic grade | I | 11 (4%) | 4 (2%) |
| | II | 170 (62%) | 104 (48%) |
| | III | 95 (34%) | 106 (49%) |
| | IV | 0 | 4 (2%) |
| Perineural invasion | Yes | 47 (17%) | 82 (38%) |
| | No | 229 (83%) | 136 (62%) |
| Venous invason | Yes | 68 (25%) | 67 (31%) |
| | No | 208 (75%) | 151 (69%) |
| Pre-operative CEA[a] | 0–3.1 $ng\,ml^{-1}$ | 147 (57%) | 90 (43%) |
| | 3.2–7.0 $ng\,ml^{-1}$ | 62 (24%) | 46 (22%) |
| | 7.1+ $ng\,ml^{-1}$ | 51 (20%) | 72 (35%) |
| Age[b] | | 64.6±9.7 years | 63.6±9.8 years |
| Maximum tumour diameter[b] | | 6.0±5.8 cm | 5.7±3.9 cm |

[a]Analysis group only (see text); [b]Mean±1 s.d.

The analysis group consisted of all randomized patients with recorded values of all relevant parameters for the analysis of prognostic factors. Sixteen Dukes' B and ten Dukes' C patients were excluded from the proportional hazards analysis because no CEA measurement was recorded within the time interval from one week before to one week after the operation. The group submitted to regression analysis thus consisted of 260 Dukes' B and 208 Dukes' C tumours.

*Statistical method*

The effect of single variables on survival was investigated by single-parameter analyses using the product-limit estimate of the survivorship function (Kaplan & Meier, 1958) and the log-rank test for statistical significance (Mantel, 1966). Estimated 5-year survival is specified ± the standard error of the estimate. A significance level of 0.05 was employed.

A multivariate regression analysis of survival data was performed using the Cox proportional hazards model (PHM) (Cox, 1972) implemented as a FORTRAN program based on the code given by Kalbfleisch and Prentice (1980). The proportional hazards assumption was tested graphically. A more detailed description of the procedure has been given recently (Bentzen *et al.*, 1988).

The covariates, i.e., the clinical and histopathological parameters to be tested for their prognostic value, were selected based on the experience from published prognostic studies (Chapuis *et al.*, 1985; Sugarbaker *et al.*, 1985). Inclusion of covariates in the regressional model was done in a step-wise manner guided by single parameter analyses. After establishing a basic model including perineural and venous invasion, tumour localization and pre-operative CEA, additional covariates were added one by one to test if any of these had significance. Covariates with a $P$-value $<0.1$ were included in the model if prior knowledge indicated their possible prognostic significance.

The endpoint used was death with cancer. Patients still alive at the time of analysis (September 1986) and patients dying from intercurrent disease with no evidence of cancer at autopsy were treated as censored observations (Table II). All other patients were considered dead from cancer, thus obtaining a conservative estimate of the frequency of cancer deaths. No patient was lost for follow-up. Test of significance as well as the multivariate analysis were based on the full observation time available (i.e., up till 7 years).

*Post-operative radiotherapy*

All patients were treated with 8–16 MV photons in the prone position either with a three-field technique using one posterior and two parallel opposing lateral wedged fields or with a four-field technique using two parallel opposing lateral fields and two parallel opposing anterior–posterior fields. All fields were treated in each treatment session. The target volume specified by the CRES protocol included the pelvic cavity with the proximal field limit at the mid-level of the 5th lumbar vertebra. The distal limit was the lower margin of the obturator foramen in patients having anterior and low anterior resection, whereas in patients having abdominoperineal resection the distal limit included the perineal region. Laterally, the pelvic brim was included with a margin of 1.5 to 2 cm. The lateral fields included the posterior surface of the symphysis and the whole sacral cavity with proximal and distal limits matching those of the posterior field. Individually shaped shielding blocks were made to encompass the target defined on conventional simulator radiographs with a 2–3 cm margin.

A total target dose of 50 Gy was given with 2 Gy/fraction, 5 days a week. The irradiation was given as a split-course,

**Table II** Carcinoma status at time of death/censoring

| | *Dukes' B* | *Dukes' C* |
|---|---|---|
| Dead with verified carcinoma | 59 (21%) | 110 (50%) |
| Dead – no autopsy | 10 (4%) | 12 (6%) |
| Dead – no carcinoma at autopsy[a] | 10 (4%) | 9 (4%) |
| Still alive[a] | 197 (71%) | 87 (40%) |

[a]Treated as censored observations.

with a 2 week break after 30 Gy. Radiotherapy was started within 30 days after surgery or in patients with surgical complications within 60 days after surgery.

A random sample of 45 simulator films and port films were reviewed by the radiotherapy group. In all but two cases (96%) the target volume was judged to be adequately treated. In one patient, the perineal region was erroneously excluded from the field; in the other the margin to the sacral bone was insufficient (~0.5 cm).

### Results

Figure 1 shows the product-limit estimate of the survivorship functions for Dukes' B and Dukes' C patients with or without post-operative radiotherapy. The overall 5-year actuarial survival was 68.9±4.6% in Dukes' B patients randomized to receive radiotherapy (+RT) and 71.4±4.7% in those randomized to surgery alone (−RT) the difference being insignificant ($\chi_1^2=0.83$, $P=0.36$). In Dukes' C patients the 5-year survival was 36.3±5.3% (+RT) and 23.4±6.0% (−RT). Again the difference was not statistically significant ($\chi_1^2=0.01$, $P=0.92$).

Single parameter analyses were performed to establish an appropriate scoring of age (Table III) and tumour size (Table IV) for the PHM analysis.

In the Dukes' B group old patients (age above 70) had a significantly poorer prognosis than younger patients. A linear test-for-trend based on the log-rank test (Tarone, 1975) yielded a highly significant $P$ value ($\chi_1^2=11.2$, $P=0.0008$). Increasing tumour size evaluated as the maximum diameter of the tumour seemed to be associated with an improved prognosis, but a linear test-for-trend yielded a non-significant $P$ value of 0.11 ($\chi_1^2=2.55$).

Age had no significant prognostic value in patients with Dukes' C tumours (test-for-trend $\chi_1^2=0.0$, $P=1.0$). All 10-year age groups had comparable 5-year survival around 30% and median surival around 3 years. Increasing tumour size seemed to worsen prognosis, but again the test-for-trend was non-significant ($\chi_1^2=2.71$, $P=0.10$).

### *Regression analysis*

Patients with Dukes' B and Dukes' C tumours were analyzed as two separate groups. Although this strategy may lead to some loss of statistical power compared to the technically feasible stratified PHM analysis (Kalbfleisch & Prentice, 1980), it was preferred here in order to avoid the assumption about identical regression coefficients for the two stages. A partial motivation for this choice is the apparent interaction between age and stage (Table III) and tumour size and stage (Table IV) where opposite trends are seen in the two stages.

**Figure 1** Kaplan–Meier estimate of survival after adjuvant radiotherapy and resection alone in 276 patients with Dukes' B and 218 patients with Dukes' C tumours.

**Table III** 5-year survival according to age-group

| | *Dukes' B*[a] | | *Dukes' C* | | |
|---|---|---|---|---|---|
| | *5-year survival*[b] | *No. of patients* | *5-year survival*[c] | *No. of patients* | *Median (years)* |
| 20–50 | 81.6±8.1% | 22 | 24.7±11.9% | 21 | 3.16 |
| 51–60 | 84.3±5.1% | 56 | 11.4± 7.1% | 50 | 3.25 |
| 61–70 | 74.4±4.9% | 117 | 35.0± 5.9% | 89 | 3.06 |
| 71–80 | 49.3±7.0% | 81 | 37.2± 7.5% | 58 | 3.32 |

[a]Median survival not evaluable; [b]Test-for-trend ($\chi_1^2=11.2$, $P=0.0008$); [c]Test-for-trend ($\chi_1^2=0.0$, $P=1.0$).

Such interactions may be included in the modelling but will produce a more complex final model. The scoring of covariates is given in Table V. Graphical tests showed that the proportional hazards assumption was reasonable. Results for each covariate are briefly presented here.

Patient age was entered in the PHM both as a categorical and as a continuous variable. Age had no prognostic value in the Dukes' C group. In the Dukes' B group, patients aged above 60 years experienced an increasing hazard with increasing age. The age dependence was highly significant ($P=0.0004$). In patients below 60 years of age no correlation was found between age and prognosis.

The male/female ratio was 1.28 among the patients with Dukes' B tumours. Sex had no prognostic value in this group ($P=0.25$). In Dukes' C patients, a male/female ratio of 0.90 was found. Sex was included in the final regression model with a $P$ value of 0.07 because other authors (e.g., Chapuis *et al.*, 1985) have found this to be a prognostic factor. Females had a better prognosis than males.

Anatomical location of the primary tumour may be characterized in different (highly correlated) ways, e.g., whether the tumour is located in the rectum or the colon, or by the

**Table IV** 5-year survival according to tumour size

| | *Dukes' B*[a] | | *Dukes' C* | | |
|---|---|---|---|---|---|
| | *5-year survival*[b] | *Patients* | *5-year survival*[c] | *Median (years)* | *Patients* |
| 0–4 cm | 64.4±5.7% | 101 | 41.3±7.2% | 4.02 | 78 |
| 5–7 cm | 73.0±4.9% | 120 | 31.6±6.4% | 3.32 | 94 |
| 8+ cm | 74.7±6.4% | 55 | 16.8±6.0% | 2.61 | 46 |
| (9+ cm | 81.5±7.3% | 28) | | | |

[a]Median survival not evaluable; [b]Test-for-trend ($\chi_1^2=2.55$, $P=0.11$); [c]Test-for-trend ($\chi_1^2=2.75$, $P=0.10$).

**Table V** Scoring of covariates in PHM analysis

| *Covariate* | *Scores* | |
|---|---|---|
| Age[a] | Actual age if ≧60 y. Set equal to 60 y if age <60 y | |
| Sex | 1: Male | 0: Female |
| Localization[b] | 1: dist. ≦10 cm | 0: dist. >10 cm |
| Complicating disease | 1: Yes | 0: No |
| Resection of neighbour organs | 1: Yes | 0: No |
| Histologic grade[c] | 1: IV+III | 0: I+II |
| Perineural invasion | 1: Yes | 0: No |
| Venous invasion | 1: Yes | 0: No |
| Max. diameter | Actual diameter in cm | |
| CEA level: 2: 7.1+ng $ml^{-1}$, 1: 3.2–7.0 ng $ml^{-1}$, 0: 0–3 ng $ml^{-1}$. | | |

[a]In addition age ≦50 y was tested as a prognostic factor; [b]Measured as the tumour's distance from the anal verge; [c]Alternative scoring tested: 4: IV, 3: III, 2: II, 1: I.

location of the tumour relative to the peritoneal reflection. Here the distance from the anal verge, evaluated by rectoscopy, was used. A distance >10 cm was associated with an improved prognosis in both Dukes' B and C patients ($P$=0.005 and $P$=0.018, respectively).

Tumour size, evaluated as the largest linear diameter of the tumour, was not a statistically significant prognostic factor in Dukes' B tumours. In the Dukes' C tumours increasing tumour size led to a worsening of the prognosis ($P$=0.018). Tumour size in 3 directions was available, but the maximal diameter had the same prognostic value as the area or volume measures.

While insignificant in the Dukes' B group ($P$=0.39), the necessity of neighbour organ resection was associated with a poor prognosis in Dukes' C patients ($P$=0.06).

Complicating disease, i.e., the presence of conditions assumed to influence the prognosis – but not directly tumour related – was also tested in the analysis. This covariate had no statistical significance.

Three histopathological parameters were available for analysis. Perineural and venous invasion, and histological grading. Perineural invasion had very strong prognostic significance in both groups. Both venous and perineural invasion were strong prognosticators in Dukes' C tumours. When survival was corrected for these two factors, the histologic grade had no significant influence on survival ($P$=0.16). In Dukes' B tumours a relatively close correlation ($r$=0.39 estimated from the empirical covariance matrix) was found between venous invasion and histological grade. In view of the importance of venous invasion in Dukes' C disease, this covariate was given the higher priority and included in the final model.

Pre-operative CEA level was classified in 3 groups; 0–3.1 ng $ml^{-1}$, 3.2–7.0 ng $ml^{-1}$ and 7.1+ ng $ml^{-1}$. The cutpoints were chosen to be the median (3.1 ng $ml^{-1}$) and the 75th percentile (7.1 ng $ml^{-1}$) of the CEA values in the combined set of Dukes' B and C patients. The hazard rate was found to increase with increasing CEA. In Dukes' B tumours, a patient with a plasma CEA above 7.1 ng $ml^{-1}$ (20% of the Dukes' B cases) had a 1.92 times as high hazard rate as a patient in the 0–3.1 ng $ml^{-1}$ group. Similarly, a Dukes' C patient with CEA in the highest group (35% of the Dukes' C cases) had a 1.51 times as high hazard rate as a patient in the low CEA group.

Radiotherapy was added as a covariate in the final regression model. In Dukes' B patients the $P$ value for this covariate was 0.23. The estimated hazard of a patient having radiotherapy was 1.20 times that of a patient having surgery only. In Dukes' C patients the $P$ value of the covariate radiotherapy was 0.46. The estimated hazard of a patient receiving radiotherapy was 1.02 times that of a patient having surgery only.

The final regression parameters are presented in Table VI.

**Table VI** Final regression model

| *Covariate* | *B* | *s.d.* | *Exp(B)* | *P value* |
|---|---|---|---|---|
| *Dukes' B* | | | | |
| Perineural invasion | 0.769 | 0.286 | 2.158 | 0.004 |
| Tumour localization | 0.647 | 0.253 | 1.909 | 0.005 |
| Venous invasion | 0.398 | 0.266 | 1.488 | 0.068 |
| CEA level | 0.327 | 0.148 | 1.387 | 0.014 |
| Age above 60 | 0.071 | 0.021 | 1.074 | 0.0004 |
| *Dukes' C* | | | | |
| Perineural invasion | 0.535 | 0.195 | 1.708 | 0.003 |
| Resection other organs | 0.444 | 0.289 | 1.559 | 0.062 |
| Venous invasion | 0.410 | 0.196 | 1.507 | 0.018 |
| Tumour localization | 0.393 | 0.187 | 1.482 | 0.018 |
| Sex | 0.284 | 0.194 | 1.328 | 0.072 |
| CEA level | 0.207 | 0.107 | 1.230 | 0.026 |
| Max. diameter | 0.039 | 0.018 | 1.039 | 0.018 |

### *The prognostic index*

From the final regression model individual prognostic forecasts may be calculated based on a patient's prognostic index, i.e., the set of values of the significant prognostic factors. As an example Table VII presents the estimated 5-year survival for each of four hypothetical patients. These four patients are chosen to have very favourable/unfavourable prognosis in Dukes' B and C respectively. Figure 2 shows

**Figure 2** Range of prognoses predicted by the proportional hazards model in patients with Dukes' B and Dukes' C tumours. The patient characteristics of these four hypothetical cases are given in **Table VII**.

**Table VII** Prognostic indices

| | |
|---|---|
| Dukes' B | *Good prognosis:* No perineural or venous invasion, CEA≦3.1 ng $ml^{-1}$, <60 years of age, tumour located more than 10 cm from anal verge. Estimated 5-year survival 90%. |
| | *Poor prognosis:* Perineural and venous invasion, CEA≧7.1 ng $ml^{-1}$, 70 years of age, tumur less than 10 cm from anal verge. Estimated 5-year survival 9%. |
| Dukes' C | *Good prognosis:* Female, no perineural or venous invasion, CEA≦3.1 ng $ml^{-1}$, tumour diameter 5 cm, no resection of neighbour organs, tumour more than 10 cm from anal verge. Estimated 5-year survival 62%. |
| | *Poor prognosis:* Male, with perineural or venous invasion, CEA≧3.1 ng $ml^{-1}$, tumour diameter 10 cm, resection of neighbour organs, tumour less than 10 cm from anal verge. Estimated 5-year survival 0%. |

the estimated survival functions for the same four cases. While the most unfavourable cases represent extremes in the patient population it is interesting that the best prognostic characteristics were quite frequent. Among the Dukes' B and C patients 21% and 9%, respectively, had the most favourable prognostic index. Estimation of 5-year survival for patients with other prognostic indices is discussed in the Appendix.

In an attempt to look for a possible survival benefit from radiotherapy among patients with a short life expectancy i.e., with a high risk of cancer death the Dukes' B and C patients with a relative hazard above the median were selected. In these high-risk groups, the survival with and without post-operative radiotherapy was estimated. The results are given in Table VIII. No significant differences were found.

### Discussion

Dukes' stage is a well-established and very strong prognostic factor. In the single-parameter analyses Dukes' stage had stronger influence on prognosis than any other single factor. However, the prognostic models derived from this analysis predict a very broad range of prognoses within each stage. Indeed, prognostically good Dukes' C patients are likely to do better than prognostically poor Dukes' B patients. Thus, the regression models suggest very important differences in prognosis in patients with identical Dukes' stage.

In assessing the prognostic significance of patients' age as demonstrated in Dukes' B cancer in this analysis, it should be kept in mind that the survival analysis uses death with cancer as the endpoint. Thus, the survival calculated here may be expected to be lower than a standard relative survival. Still, it is interesting that the present worsening of prognosis with increasing age contradicts the findings by Block and Enker (1971) in rectal cancer and by Jensen *et al.* (1970) in colon cancer. As pointed out by Sugarbaker *et al.* (1985) the improved prognosis with increasing age observed by these authors may be partly explained by the observation by Dukes and Bussey (1958) that lower grade of malignancy is associated with older age. In a multivariate analysis this confounding effect will be corrected for, but in the current series prognosis worsened with increasing age also in the single-parameter analysis (Table III). Interestingly, the multivariate analysis by Chapuis *et al.* (1985) showed the same result as our analysis.

A poorer prognosis in young patients (below 40 years of age) could not be demonstrated in the present series. However, the number of young patients was low, implying a low power of the test.

Sex had a prognostic value in the series by Chapuis *et al.* (1985). However, in the present analysis, this result was not reproduced in Dukes' B tumours, and in Dukes' C tumours, sex was the least important of the parameters in the model.

Among the clinical variables, the distance of the tumour from the anal verge is important in both stages. This is probably a consequence of difficulty in securing a tumour free lateral resection margin in low situated tumours. (Quirke *et al.*, 1986), but a difference in lymphatic drainage between low and high situated tumours may also influence the prognosis (Sugarbaker *et al.*, 1985).

A number of authors have failed to demonstrate a clear relationship between tumour size and prognosis. Spratt and Spjut (1967) found a slight tendency of very large tumours to have an improved prognosis. The same trend (although not statistically significant at the 5% level) seems to be found in the Dukes' B tumours in this series. However, after correction for other patient characteristics, tumour size has no prognostic value. In patients with large Dukes' C tumours a tendency towards diminished 5-year-survival is observed. This tendency becomes significant in the regression model. The fact that the maximum tumour diameter has the same discriminative power as area or volume is in accordance with the findings by Miller *et al.* (1985).

Recently, Wolmark *et al.* (1986) analyzed the prognostic value of three proposed modifications of the classification of Dukes' C colorectal cancer, *viz.* the level of histologically positive nodes, the tumour preparation depth, and the number of histologically positive nodes. The number of positive nodes was the strongest prognostic discriminate. No attempt was made to correct for the effect of other clinical or histopathological variables. Unfortunately, the number of histologically positive nodes was not available in our analysis. However, the prognostic index suggested by the present analysis has higher discriminative power than the number of positive lymph nodes had in the series of Wolmark *et al.* (1986).

Perineural invasion had the strongest impact on survival in both stages. This is in agreement with Seefeld and Bargen (1943) who found an increased rate of local recurrences as well as a decreased 5-year survival in patients with perineural invasion. Also venous invasion is known to have prognostic value (Grinnell, 1950). When both perineural and venous invasion were entered into the PHM the first turned out to have the strongest prognostic influence.

In addition to being the result of a subjective evaluation, histologic grade is known to be strongly correlated with stage in colorectal cancer (Newland *et al.*, 1981), and its independent prognostic value is questionable. Nevertheless, Chapuis *et al.*, (1985) performed a PHM analysis of prognostic factors in 709 patients with Dukes' A, B, C, or D colorectal carcinoma in which clinico-pathological stage was found to be the strongest prognostic factor, followed by age, histologic grade, venous invasion, sex, direct spread and the presence of obstruction. Perineural invasion and CEA were not included in the analysis.

Jass *et al.* (1986) also applied the Cox PHM to establish a histopathological grading system in rectal cancer. Seven-grade related parameters were entered in a survival analysis of 447 cases. Lymphocytic infiltration, tubule configuration and pattern of growth were the strongest prognosticators. When stage-related parameters were entered into the model, lymphocytic infiltration, number of involved nodes and spread through the bowel wall were the most important parameters. It should be noted that Dukes' stage was excluded from the model because of its composite nature. In the current study the intent was to refine rather than to replace Dukes' classification.

Diverse conclusions have been made concerning the survival advantage of lymphocytic infiltration (e.g., Svennevig *et al.*, 1984; Thynne *et al.*, 1980) and the exact mechanism behind an eventual positive influence on survival remains unclear (Jass *et al.*, 1986).

Elevated pre-operative CEA has been shown to be correlated with time to recurrence, in Dukes' B and C cancer (Wanebo, 1978), but has also been shown to be correlated with other clinical and histopathological factors. The present multivariate analysis demonstrates an independent effect on survival of the pre-operative CEA level. Among the 20% of Dukes' B and the 35% of Dukes' C patients with CEA concentration above 7.1 ng ml$^{-1}$ the hazard rate is 1.92 and 1.51 times higher, respectively, than for patients with CEA in the normal range. In other words CEA concentration is among the strongest prognosticators investigated in this study. Furthermore, a semi-quantitative relationship seems to exist between the hazard rate and the level of CEA.

**Table VIII** 5-year and median survival in high-risk patients[a]

| | *+RT* | *−RT* | *P values*[b] |
|---|---|---|---|
| Dukes' B[c] | 58.0±7.0% | 58.8±7.0% | N.S. ($\chi_1^2=0.24$, $P=0.62$) |
| Dukes' C | 20.5±6.2% | 11.8±5.9% | N.S. ($\chi_1^2=0.71$, $P=0.40$) |
| (median) | (709 days) | (924 days) | |

[a]Defined as patients with a relative hazard above the median; [b]Significance tested by the log-rank test. [c]Median survival not evaluable.

Cross-validation of this observation in an independent series would be desirable.

In recent years, a number of immunohistochemical markers has been suggested in colorectal cancer. This paper has demonstrated the presence of very strong prognostic factors in this disease. Therefore a multivariate analysis with adequate correction for the effect of known clinical and histological prognosticators is required to assess the independent value of any proposed prognosticator.

Numerous studies using historical controls (Green, 1981; Sichy, 1982; Kopelson, 1983; Hoskins et al., 1985; Tepper et al., 1987) and the randomized study by Gerard et al. (1985) have suggested an improved local control after radiotherapy in colorectal cancer. However, the randomized study by the Gastrointestinal Study Group (1985) showed no significant difference in time to tumour recurrence between the radiation plus surgery (50 pts) and the surgery-alone (58 pts) arms. Only when chemotherapy and radiotherapy were combined in the adjuvant treatment a significant difference in time to recurrence was found. Overall survival did not differ significantly among the treatment arms. The present analysis could not demonstrate any positive effect on survival after post-operative radiotherapy. Without entering the discussion on the methodological problems of using historical controls, it is interesting to note that the lack of difference in survival between the two arms in this study seems to spring not from a poor (relative to that of other studies) result in the radiotherapy arm but rather from a good result in the surgery-alone arm. Thus, if appropriate surgery is performed, radiotherapy seems to have no positive effect on survival. Delivering the radiation as a split course may have reduced the efficacy of the treatment. However, the actual importance of overall treatment time in radiotherapy of colorectal cancer remains an open question. The relevance of radiotherapy should not be judged solely from its influence on survival. Local control is of obvious importance to the patient and is a more sensitive clinical endpoint for measuring the effect of radiotherapy. Furthermore, an analysis of local control might be of value in identifying subsets of patients in which intensified local treatment would be indicated. An analysis of this endpoint with correction for other prognostic factors awaits a revision of the database.

In conclusion, clinical and histopathological variables collected prospectively in a randomized multi-centre study of post-operative radiotherapy in Dukes' B and C colorectal cancer were evaluated with respect to their prognostic importance using death with cancer as the endpoint. From the set of available parameters a proportional hazards model was derived containing parameters with an independent, significant influence on prognosis. Based on these models a prognostic forecast may be made in individual patients with Dukes' B or C tumours. The construction of such a model is an important step towards the definition of high-risk patients requiring more aggressive adjuvant therapy. Furthermore, such prognostic models may add important knowledge to the understanding of the natural history of the disease.

Retrospective analyses of prognostic factors will often miss data on one or more clinicopathological variables of possible importance. However, such studies are very important in defining the set of parameters that ought to be included in a prospective study. There is no doubt that a complete prognostic model in colorectal cancer should include both clinical and pathological parameters together with pre-operative CEA. Several of these variables are correlated, thus allowing different subsets of these to define prognostic models with equal discriminative power. The priority given to prognostic factors should not only reflect their prognostic value but also take their inter-observer reproducibility and their mutual independence into account. Other factors influencing the priority of prognosticators are convenience to the patient, ease of measurement and the cost of measurement.

The CRES study was initiated to investigate the possible effect of post-operative radiotherapy. No such effect was demonstrated. In the present paper, the survival benefit from radiotherapy was tested with correction for known prognostic factors. No such benefit could be demonstrated. Finally, the effect of radiotherapy was tested among high risk Dukes' B and Dukes' C patients. Once again radiotherapy as administered in the CRES protocol had no positive effect on patient survival.

Although avoiding cancer death is the major goal in cancer treatment, the quality of life of the patients is another very important consideration. One remaining question is whether radiotherapy will prolong the time to the first local recurrence. An early analysis of this series suggested that this may be the case in Dukes' C tumours. However, a renewed careful analysis of the material with extended observation time cast doubt on this finding.

It is remarkable that the predicted 5-year survival rates ranged from 9% to 90% and from 0% to 62% in subgroups of patients with Dukes' B and Dukes' C tumours respectively. Although the worst prognoses represent limiting cases in the patient population, subsets of Dukes' B and C patients with very short expected lifetime have been identified in the present analysis. An increased effort towards a more aggressive adjuvant therapy in these patients is strongly needed.

Supported by grant No. 87-117 from the Danish Cancer Society and grant CA-29026 from the NCI, DHHS. We would like to thank Dr Joel Tepper for his helpful comments on this paper. Soren M. Bentzen gratefully acknowledges the hospitality and available resources of the Department of Biomathematics, The University of Texas System Cancer Center, M.D. Anderson Hospital & Tumour Institute in the preparation of this manuscript. Special thanks to Ms Connie Seifert for skilful secretarial assistance.

### Appendix: Calculation of prognostic forecasts from the regression model

The prognostic best cases in **Table VII** may be used as reference cases for calculating the prognosis in patients with other prognostic indices. This is accomplished using the regression parameters in **Table VI** and the estimated 5-year survival for the prognostic most favourable cases from **Table VII**. The estimated survival should be taken as a fraction of one, i.e., 0.90 for Dukes' B and 0.62 for Dukes' C. There are three steps in the procedure:

First, write down the values of the covariates for the actual case using the codes from **Table V**. Only age (Dukes' B) and maximum tumour diameter (Dukes' C) require a slightly different procedure. The covariate describing the prognostic effect of age is set equal to zero for patients below 60 years of age, and equal to the actual age minus 60 years for patients above 60 years of age (e.g., equal to 13 for a 73-year-old patient). The covariate describing the effect of tumour size is set equal to the maximum tumour diameter measured in cm minus 5 cm (the reference size for the best case in **Table VII**), e.g., equal −3 for a 2 cm diameter tumour.

Second, multiply the value of each covariate with the corresponding B-value from **Table VI** and add together all these products. Let $s$ denote the resulting sum.

Third, calculate exp($s$) and raise the 5-year survival from **Table VII** to this power. The result is the estimated survival for the actual case, again expressed as a fraction of one.

*Example.* Calculate the estimated 5-year survival for a 65-year-old patient with a Dukes' B tumour, a pre-operative CEA level of 8 ng ml$^{-1}$ and with venous invasion. There is no perineural invasion and the tumour is located more than 10 cm from the anal verge. *Solution:* First note that there is no contribution from the covariates describing perineural invasion and localization as these agree with the values for the prognostic best case. The covariate for venous invasion is equal to one, for age the covariate is equal to 5 (actual age minus 60 years), and for CEA it is 2. Thus $s$ is

$$s = 0.398 \cdot 1 + 0.071 \cdot 5 + 0.327 \cdot 2 = 1.407$$

and exp($s$) becomes 4.08. The estimated 5-year survival for the actual case is then the 5-year survival for the prognostic best case raised to the power 4.08, i.e., $(0.90)^{4.08} = 0.65$ or 65%.

### References

BALSLEV, I., PEDERSEN, M., TEGLBJAERG, P.S. & 12 others (1986). Postoperative radiotherapy in Dukes' B and C carcinoma of the rectum and rectosigmoid. A randomized multicenter study. *Cancer*, **58**, 22.

BALSLEV, I., PEDERSEN, M., TEGLBJAERG, P.S. & 11 others (1982). Postoperative radiotherapy in rectosigmoid cancer Dukes' B and C: Interim report from a randomized multicenter study. *Br. J. Cancer*, **46**, 551.

BENTZEN, S.M., POULSEN, H.S., KAAE, S. & 5 others (1988). Prognostic factors in osteosarcomas. A regression analysis. *Cancer*, **62**.

BLOCK, G.E. & ENKER, W.E. (1971). Survival after operations for rectal carcinoma in patients over 70 years of age. *Ann. Surg.*, **174**, 521.

CHAPUIS, P.H., DENT, O.F., FISHER, R. & 4 others (1985). A multivariate analysis of clinical and pathological variables in prognosis after resection of large bowel cancer. *Br. J. Surg.*, **72**, 698.

COX, D.R. (1972). Regression models and life tables. *J. Roy. Statist. Soc B*, **34**, 187.

DUKES, C.E. & BUSSEY, H.J.R. (1958). The spread of rectal cancer and its effect on prognosis. *Br. J. Surg.*, **12**, 309.

GASTROINTESTINAL TUMOR STUDY GROUP (1985). Prolongation of the disease-free interval in surgically treated rectal carcinoma. *N. Engl. J. Med.*, **312**, 1465.

GERARD, A., BERROD, J.-L., PENE, F. & 9 others (1985). Interim analysis of a phase III study on preoperative radiation therapy in resectable rectal carcinoma. Trial of the Gastrointestinal Tract Cancer Cooperative Group of the European Organization for Research on Treatment of Cancer (EORTC). *Cancer*, **55**, 2373.

GREEN, J.P. (1981). Rectal cancer: Adjuvant radiation therapy. *Front. Radiat. Ther. Oncol.*, **15**, 102.

GRINNELL, R.S. (1950). Lymphatic metastases of carcinoma of the colon and rectum. *Ann. Surg.*, **131**, 494.

HOSKINS, R.B., GUNDERSON, L.L., DOSERETZ, D. & GALDABINI, J. (1985). Adjuvant postoperative radiotherapy in carcinoma of the rectum and the rectosigmoid. *Cancer*, **55**, 61.

JASS, J.R., ATKIN, W.S., CUZICK, J. & 4 others (1986). The grading of rectal cancer: Historical perspectives and a multivariate analysis of 447 cases. *Histopathology*, **10**, 437.

JENSEN, H.E., NIELSEN, J. & BALSLEV, I. (1970). Carcinoma of the colon in old age. *Ann. Surg.*, **171**, 107.

KALBFLEISCH, J.D. & PRENTICE, R.L. (1980). The statistical analysis of failure time data. J. Wiley and Sons: New York.

KAPLAN, E. & MEIER, P. (1958). Nonparametric estimation from incomplete observations. *J. Amer Statist. Assoc.*, **53**, 457.

KOPELSON, G. (1983). Adjuvant postoperative radiation therapy for colorectal carcinoma above the peritoneal reflection. *Cancer*, **51**, 102.

KRONBORG, O., FENGER, C., BERTELSEN, K. & 11 others (1988). Escape clauses in a multicentre trial. Unforeseen problems and their potential influence on the general validity of the conclusions. *Theoret. Surg.* **2**, 157.

MANTEL, N. (1966). Evaluation of survival data and two new rank order statistics arising in its consideration. *Cancer Chemother. Rep.*, **50**, 163.

MILLER, W., OTA, D., GIACCO, G. & 4 others (1985). Absence of relationship of size of primary colon carcinoma with metastasis and survival. *Clin. Expl. Metastasis*, **3**, 189.

NEWLAND, R.C., CHAPUIS, P.H., PHEILS, M.T. & MACPHERSON, J.G. (1981). The relationship of survival to staging and grading of colorectal carcinoma: A prospective study of 503 cases. *Cancer*, **47**, 1424.

QUIRKE, P., DURDEY, P. DIXON, M.F. & WILLIAMS, N.S. (1986). Local recurrence of rectal adenocarcinoma due to inadequate surgical resection. Histopathological study of lateral tumour spread and surgical excision. *Lancet*, **ii**, 996.

SEEFELD, P.H. & BARGEN, J.A. (1943). The spread of carcinoma of the rectum: Invasion of lymphatics, veins and nerves. *Ann. Surg.*, **118**, 76.

SICHY, B. (1982). The place of radiotherapy in the management of rectal adenocarcinoma. *Cancer*, **50**, 2631.

SPRATT, J.S. & SPJUT, H.J. (1967). Prevalence and prognosis of individual clinical and pathological variables associated with colorectal carcinoma. *Cancer*, **20**, 1976.

SUGARBAKER, P.H., GUNDERSON, L.L. & WITTES, R.E. (1985). Colorectal cancer. In *Cancer. Principles and Practice of Oncology*, DeVita, S. *et al.* (eds) Vol. 1, p. 795. J.B. Lippincott Company: Philadelphia.

SVENNEVIG, J.L., LUNDE, O.C., HOLTER, J. & BJORGSVIK, D. (1984). Lymphoid infiltration and prognosis in colorectal carcinoma. *Br J. Cancer*, **49**, 375.

TARONE, R.E. (1975). Tests for trend in life table analysis. *Biometrika*, **62**, 679.

TEPPER, J.E., COHEN, A.M., WOOD, W.C., ORLOW, E.L. & HEDBERG, S.E. (1987). Postoperative radiation therapy of rectal cancer. *Int. J. Radiat. Oncol. Biol. Phys.*, **13**, 5.

THYNNE, G.S., WEILAND, L.H., MOERTEL, C.G. & SILVERS, A. (1980). Correlation of histopathologic characteristics of primary tumour and uninolved regional lymph nodes in Dukes' C colonic carcinoma with prognosis. *Mayo Clinic Proc.*, **55**, 243.

WANEBO, H.J., RAW, B., PINSKY, C. & 4 others (1978). Preoperative carcinoembryonic antigen level as a prognostic indicator in colorectal cancer. *N. Engl. J. Med.*, **299**, 448.

WOLMARK, N., FISHER, B. & WIEAND, H.S. (1986). The prognostic value of the modifications of the Dukes' C class of colorectal cancer. An analysis of the NSABP clinical trials. *Ann. Surg.*, **203**, 115.